# Exploring microstructure and petrophysical properties of microporous volcanic rocks through 3D multiscale and super-resolution imaging

**DOI:** 10.1038/s41598-023-33687-x

**Published:** 2023-04-24

**Authors:** Gianmarco Buono, Stefano Caliro, Giovanni Macedonio, Vincenzo Allocca, Federico Gamba, Lucia Pappalardo

**Affiliations:** 1 Istituto Nazionale di Geofisica e Vulcanologia, Osservatorio Vesuviano, Naples, Italy; 2 Department of Earth, Environmental and Resources Sciences, University of Naples Federico II, Naples, Italy; 3 Thermo Fisher Scientific, Bordeaux, France

**Keywords:** Geology, Petrology, Volcanology

## Abstract

Digital rock physics offers powerful perspectives to investigate Earth materials in 3D and non-destructively. However, it has been poorly applied to microporous volcanic rocks due to their challenging microstructures, although they are studied for numerous volcanological, geothermal and engineering applications. Their rapid origin, in fact, leads to complex textures, where pores are dispersed in fine, heterogeneous and lithified matrices. We propose a framework to optimize their investigation and face innovative 3D/4D imaging challenges. A 3D multiscale study of a tuff was performed through X-ray microtomography and image-based simulations, finding that accurate characterizations of microstructure and petrophysical properties require high-resolution scans (≤ 4 μm/px). However, high-resolution imaging of large samples may need long times and hard X-rays, covering small rock volumes. To deal with these limitations, we implemented 2D/3D convolutional neural network and generative adversarial network-based super-resolution approaches. They can improve the quality of low-resolution scans, learning mapping functions from low-resolution to high-resolution images. This is one of the first efforts to apply deep learning-based super-resolution to unconventional non-sedimentary digital rocks and real scans. Our findings suggest that these approaches, and mainly 2D U-Net and pix2pix networks trained on paired data, can strongly facilitate high-resolution imaging of large microporous (volcanic) rocks.

## Introduction

The rapid deposition and lithification of volcanic products during large explosive eruptions originate significant volumes of microporous rocks, typically in the form of tuffs (i.e., consolidated pyroclasts). They are hence usually widespread in volcanic areas both as surface rocks and subsurface rocks, where aquifers develop and geothermal reservoirs can be emplaced, impacting volcano dynamics and the related signals detected by monitoring networks. The study of their microstructure and petrophysical properties is thus valuable for volcanological, geothermal energy, oil and gas, hydrogeological, and other engineering (e.g., building material, nuclear waste storage, CO_2_ adsorption/capture) applications^1–8^. The rapid origin of these rocks, however, leads to complex microstructures, where pores are dispersed in a very fine, heterogeneous and lithified matrix, making their exploration challenging. Particularly, tuffs are defined as the consolidated equivalent of volcanic (fallout or flow) ash, i.e., fragments of different size (< 2 mm), nature (volcanic glass, crystals and eroded subsurface/surface rocks) and shape^9^. The most common tuffs typically arise from the emplacement of hot (up to > 600 °C), fast (up to > 300 m/s) and voluminous (up to > 1000 km^3^, covering up to > 20,000 km^2^) pyroclastic density currents, consisting of a mixture of gas and volcanic particles. Post-depositional alteration of volcanic glass can promote the formation of new minerals (e.g., zeolites, clays), further lithifying and complicating their structures^10^.

Recent technological advances allow to characterize rock texture and properties in 3D and non-destructively in the digital rock physics framework. Rock samples are scanned by X-ray microtomography (micro-CT) to obtain 3D digital rocks, that are then segmented (i.e., different phases are identified and labeled) and used to quantify microstructural parameters and estimate physical properties through several types of numerical simulations^11–15^. This permits to better investigate physical processes at different spatial (from sample/core-scale to pore-scale) and temporal (i.e., 4D imaging during in-situ or ex-situ experiments) scales, perform multiple simulations in different conditions, and preserve the samples for future analyses (particularly useful for drilling cores). However, as any imaging technology, micro-CT requires a trade-off between resolutions (or pixel size) able to properly resolve the pore space and fields of view (FoV; i.e., sample volume that can be imaged) able to guarantee the representativeness. Moreover, scanning smaller FoV at high resolution from a larger sample (e.g., rock cores), although does not involve relevant artefacts, may require excessively long scan times or hard X-rays^15^. Several works demonstrated the harmful effects that low resolutions can have on quantitative characterization of digital rocks, especially when fine textures are present (e.g., carbonate microporosity^16–19^).

In recent years, deep learning-based super-resolution approaches are rapidly expanding in the field of the computer vision, and super-resolution methods based on convolutional neural networks (CNNs) and generative adversarial networks (GANs) are proving to be particularly efficient. These approaches allow to improve the quality of a low resolution image, learning mapping functions from low resolution (LR) to high resolution (HR) images^20^. Testing the effectiveness of these methods on digital rocks with different features can be thus critical to improve digital rock physics workflows, such as to achieve large sample volume with high resolution or enhance fast low quality scans (e.g., for samples to be preserved and 4D imaging with large experimental apparatus and/or high temporal resolution). Some pioneering efforts have been done in this direction, leading to very promising results, although in most cases LR images were synthetically downsampled from HR scans and only conventional sedimentary digital rocks were employed^18,20–25^ (for details see^20,25^).

**Figure 1:**
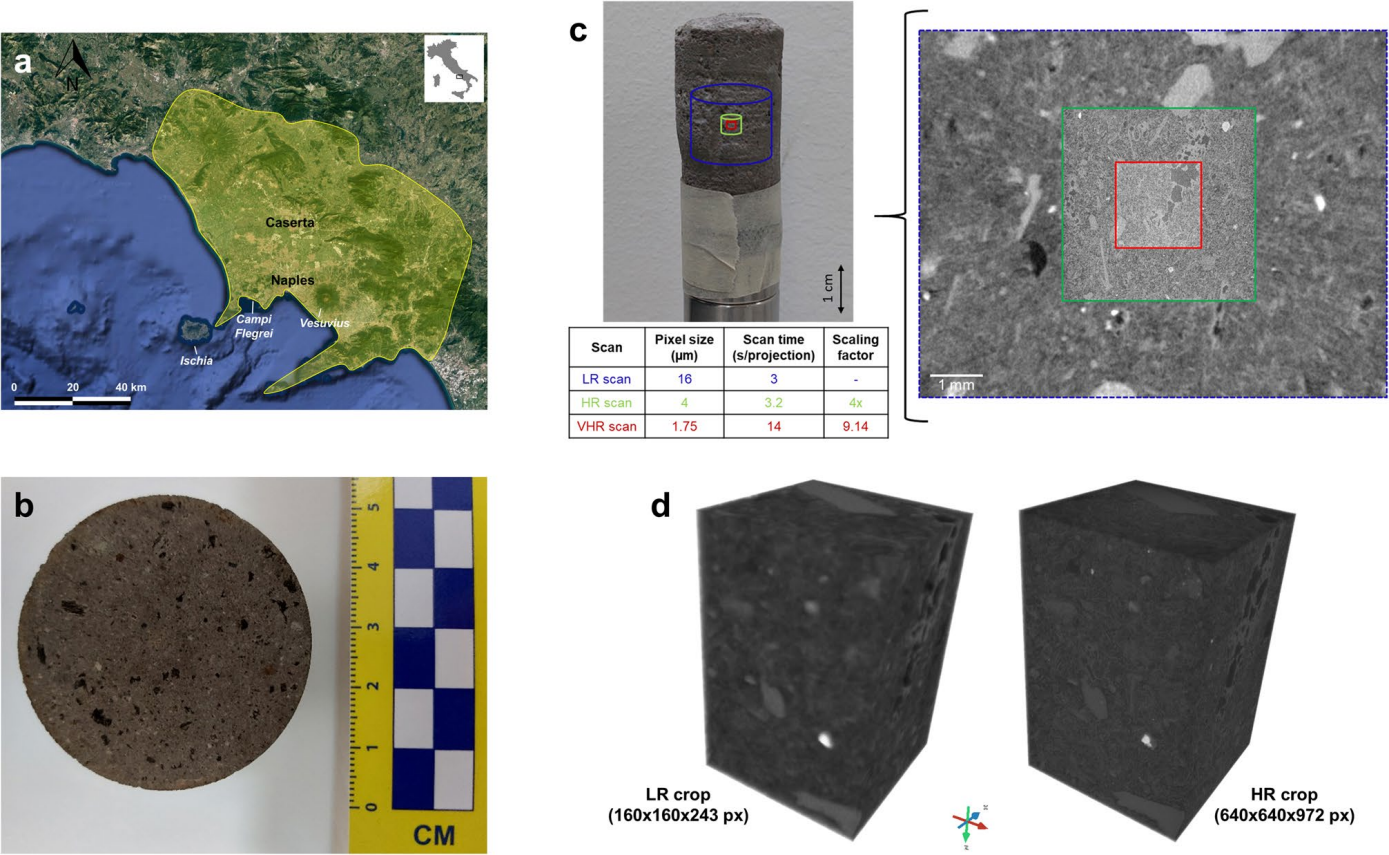
Location, distribution, aspect and 3D imaging of Campanian Ignimbrite tuff. (**a**) Area covered by Campanian Ignimbrite pyroclastic density currents on land (drawn in yellow following the constrains of Silleni et al.^26^ on map from Google Earth Pro 7.3.6: https://www.google.com/earth/about/versions/). (**b**) Cross-section of a core (diameter: 54 mm, height: 103 mm) from the Campanian Ignimbrite tuff employed for laboratory measurement. (**c**) 3D multiscale X-ray imaging. Left: Tuff core (diameter ~ 20 mm, height ~ 40 mm) acquired by X-ray microtomography. Right: 3D scans obtained progressively decreasing pixel sizes and fields of view, and increasing exposure time: low resolution (LR; 16 μm/px), high resolution (HR; 4 μm/px) and very high resolution (VHR; 1.75 μm/px) scans (XZ planes). (**d**) Low resolution (LR) image (input) and its corresponding high resolution (HR) counterpart (ground truth) used to train and validate paired super-resolutions models.

In this study we explore methods to optimize the 3D non-destructive characterization of microstructure and flow properties of microporous tuff rocks, which are “unconventional” digital rocks (i.e., largely unexplored in the digital rock physics framework). In fact, they have been poorly investigated with micro-CT so far despite their wide range of applications, possibly due to their challenging microstructures. We propose a multiscale imaging of a tuff core with progressively decreasing pixel sizes (from 16 to 1.75 μm) and fields of view, and increasing exposure time, to find a reasonable trade-off between resolution and FoV. Several 2D and 3D, CNNs- and GANs-based super-resolution approaches were then applied to real HR and LR images to further improve its imaging (avoiding synthetic downsampling to consider artefacts and problems that arise during true imaging and image registration). Particularly, networks which have shown robust results in several scientific and computer vision fields were implemented in order to effectively and quickly super-resolve these complex digital rocks. The obtained 3D images were then evaluated computing transport parameters. We investigated the Campanian Ignimbrite (CI) tuff, the dominant product of the largest Quaternary volcanic eruption in Europe, during which about 457–660 km^3^ of pyroclastic material were emitted^26^ (Fig. 1). The eruption occurred from the Campi Flegrei caldera (Naples, Italy), one of the most dangerous active volcanic area in Europe^27–29^, where surface tuffs are largely widespread and caldera-filling deposits are dominated by subsurface tuffs^5^.

## Results and discussion

Digital rock studies are a powerful tool to quantitatively explore rock microstructure and physical properties in 3D and non-destructively. Investigations mainly focused on sedimentary (especially sandstone and carbonate) rocks so far^11–14^ but they are rapidly expanding to other types of rocks, including volcanic ones^3,29–33^. Although several works are now available for unconsolidated volcanic pyroclasts and lavas with relatively simple textures, very few efforts have been done to examine consolidated microporous volcanic rocks^6,7^. Particularly, to our knowledge, their properties were never systemically and quantitatively explored in the digital rock physics framework despite their wide range of applications, possibly due to their challenging microstructures.

### Multiscale imaging

**Figure 2:**
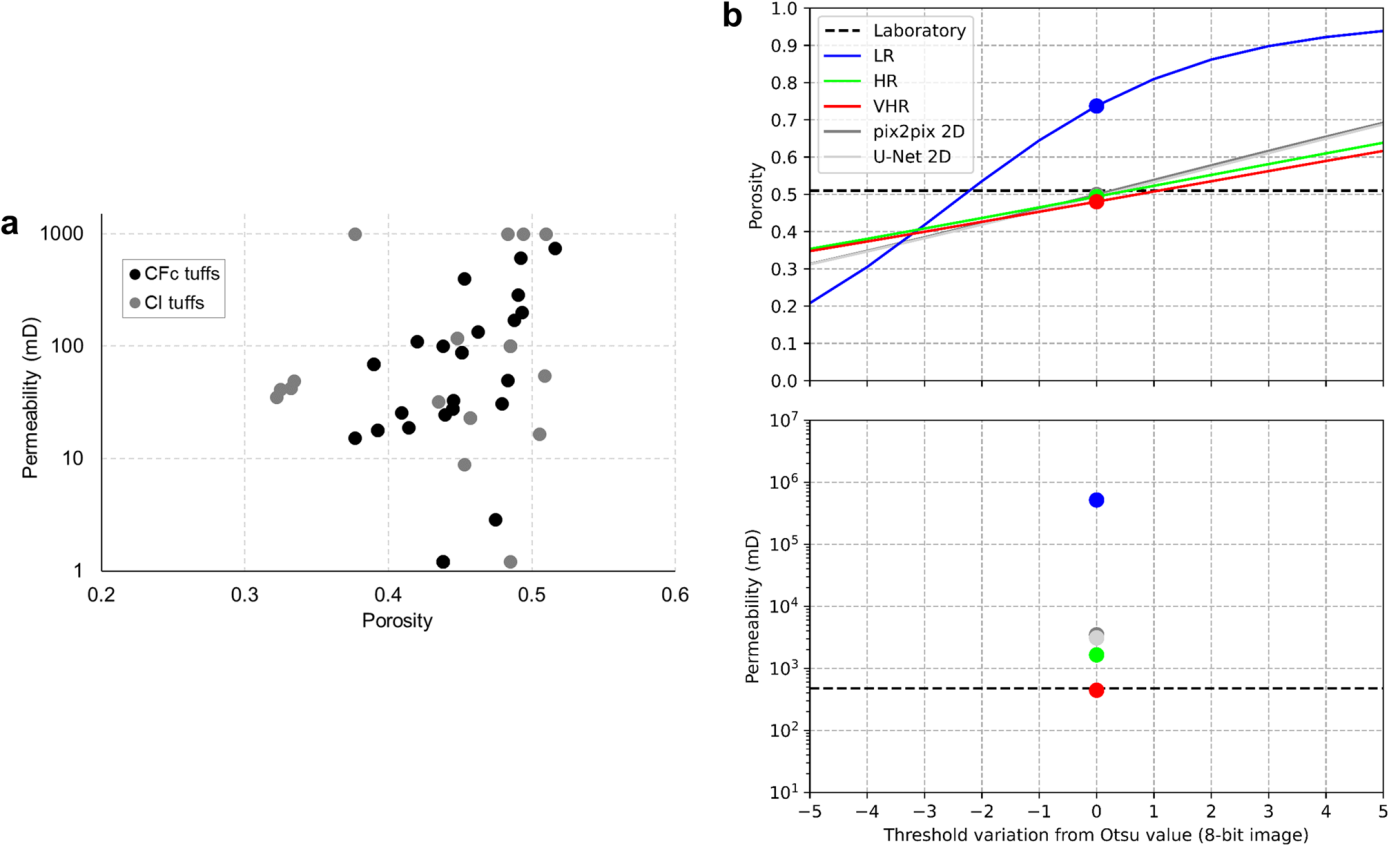
Petrophysical properties of Campi Flegrei tuffs and investigated Campanian Ignimbrite tuff. (**a**) Literature data on Campi Flegrei caldera (CFc) tuffs using conventional laboratory approaches^57–60^. Data on Campanian Ignimbrite (CI) tuff are shown in grey. CI tuff can be classified as a highly porous and moderately permeable material, whose hydraulic properties are strongly depending on composition, high pumice content and degree of welding of ignimbritic deposit. The other tuffs mainly originated during Neapolitan Yellow Tuff, Gauro, La Pietra, Nisida and Baia eruptions. (**b**) Porosity (top) and permeability (bottom) estimated through digital rock physics analyses of LR, HR and VHR images (central 640^3^ px) as well as of super-resolved images (all 2560^3^ px: 640^3^ px × scaling factor of 4, for total porosity; central 900^3^ px for intrinsic permeability, the maximum volume exploitable for our computational system, see also Fig. 6), obtained applying the best trained models (2D U-Net and pix2pix) to the LR image. The effect of threshold variations (i.e., threshold sensitivity) is also shown, estimating the total porosity as the threshold value diverges from an optimal value (i.e., Otsu value; 0 in x axis and circle symbol). Laboratory data are provided for comparison.

Microporous tuff rocks can show complex relationships between flow properties, which cannot be simply estimated obtaining classical empirical or semi-empirical (e.g., Kozeny-Carman) equations (Fig. 2a). Examining the samples of interest with specific investigations can be therefore crucial.

In order to optimize their characterization, we performed a multiscale imaging of the Campanian Ignimbrite tuff, the dominant product of the largest Quaternary volcanic eruption in Europe, widely spread in the Campi Flegrei caldera and investigated for several scientific and industrial applications^3–5,26,29^ (Fig. 1a,b). Three 3D scans (of about 1000^3^ px) were acquired by micro-CT progressively improving the resolution: LR (low resolution), HR (high resolution) and VHR (very high resolution) scans with a pixel size of 16, 4 and 1.75 μm, respectively (Fig. 1c,d). The main limitation of this technology, as for any imaging technique, is that scans with higher resolutions of a sample can be only obtained at the expense of smaller fields of view and longer scan times. Consequently, a trade-off between resolutions able to properly resolve the pore space, fields of view able to guarantee the sample representativeness and acceptable scan times to achieve a good image quality is required. One of the most reliable approaches to evaluate the efficiency of different scans is to compare their petrophysical properties with equivalent laboratory data measured following international standards^12,17,20^.

**Figure 3:**
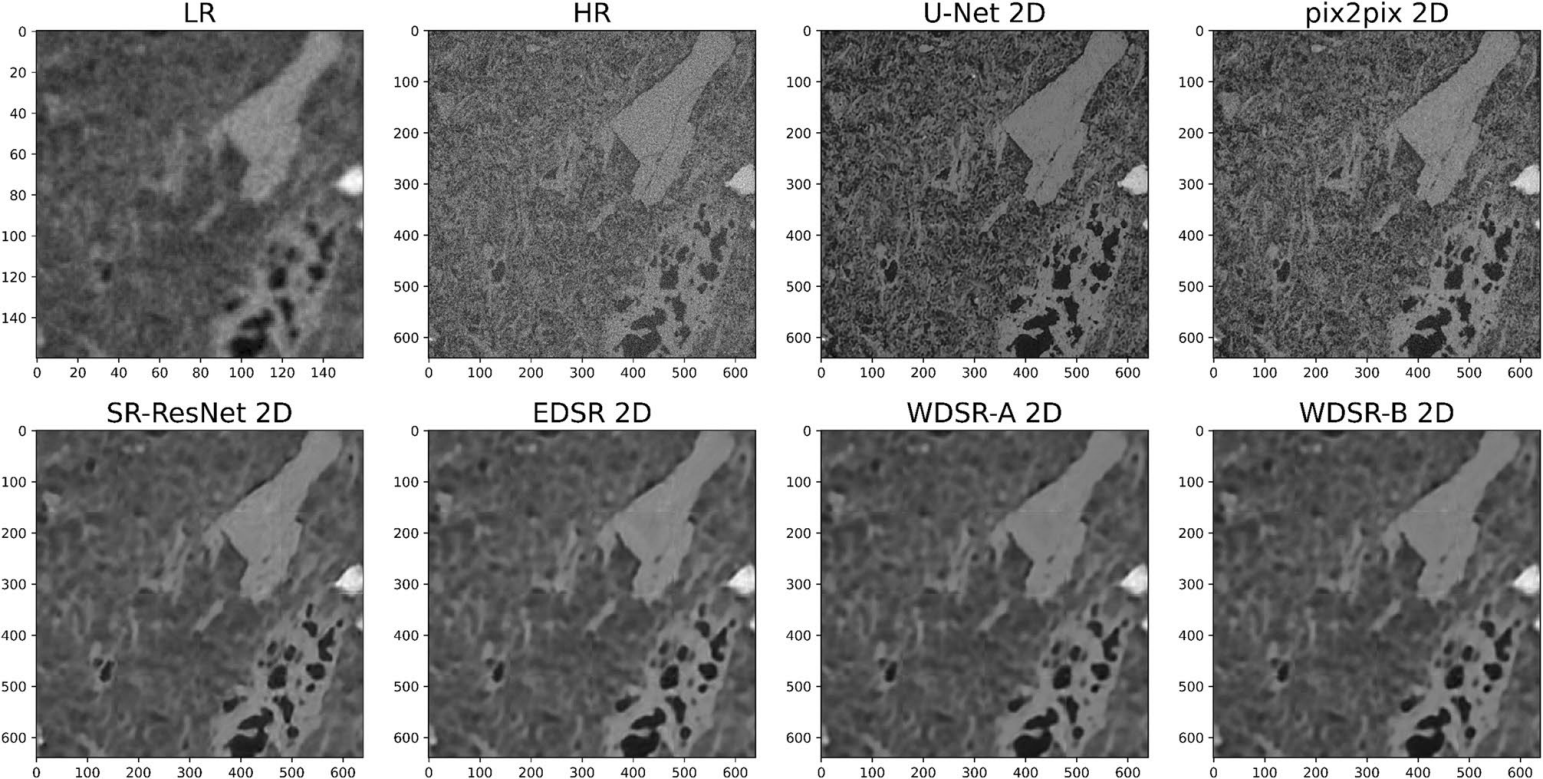
Super-resolved validation images: 2D networks trained on paired data. Validation slices for 2D CNNs (U-Net, SR-ResNet, EDSR, WDSR-a, WDSR-b) and GANs (pix2pix) employed for super-resolution imaging. LR (input) and HR (ground truth) images are also shown for comparison. Correspondent training details and image quality metrics are reported in Table 1.

**Figure 4:**
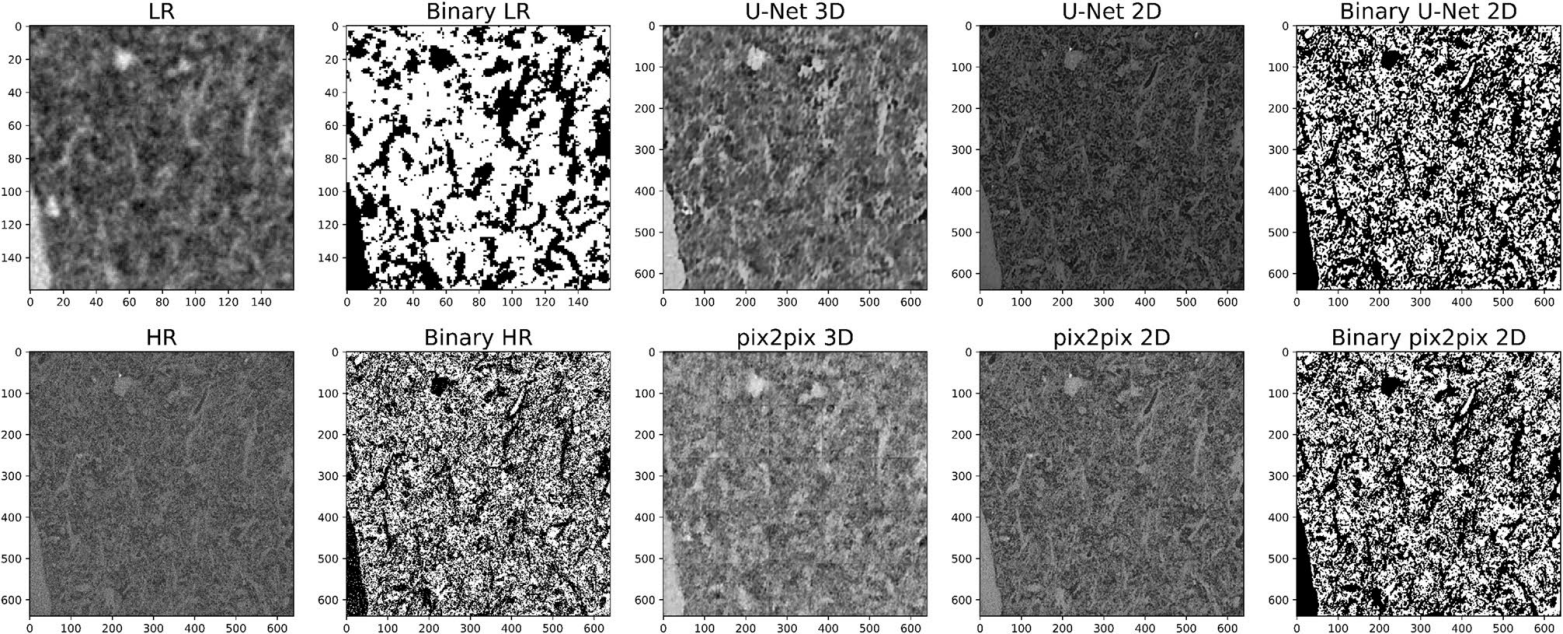
Super-resolved validation images: 2D vs. 3D networks trained on paired data. The resulting best 2D models, pix2pix and U-Net, were trained both in 2D and 3D. LR (input) and HR (ground truth) images are also shown for comparison. Correspondent training details and image quality metrics are reported in Table 1. For LR, HR and validation data from 2D networks, which outperformed the corresponding 3D models, also segmented (binary) images are provided.

We obtained a total porosity of 0.74, 0.49 and 0.48 (percolating porosity = 99%) and an intrinsic permeability of 518,021, 1649, 443 mD for our LR, HR and VHR scans, defining a simple image analysis workflow comprising segmentation with Otsu algorithm and permeability simulations with lattice Boltzmann methods on central volumes of 640^3^ px (Fig. 2b). On the other hand, an average total porosity of 0.51 and intrinsic permeability of 476 mD were obtained by laboratory measurements. Our data shows that the LR scan, although covers much of the core width, has a too high pixel size to appropriately segment the pores, leading to an overall overestimation of porosity and permeability (Fig. 2b). In detail, small pores and matrix grains are not properly discriminated due to the low resolution, resulting in portions of the segmented digital rock with overpredicted (due to prevailing pore fraction and/or very fine matrix) or underpredicted (due to prevailing solid fraction and/or very small pores) pore space (Figs. 3, 4). It is consistent with several digital rock studies on sedimentary rocks^16–19^. Conversely, HR scan has a pixel size able to properly resolve pore spaces (Figs. 3, 4) and a field of view able to guarantee the representativeness, showing petrophysical properties almost consistent with laboratory data (Fig. 2b). Finally, VHR scan allows to achieve values even closer to laboratory data despite its small field of view (Fig. 2b). We highlight that it is just a qualitative comparison between digital and laboratory measurements. Our digital results were, in fact, obtained adopting a very direct workflow to minimize the influence of different filtering, segmentation and simulation approaches. Moreover, laboratory data was measured on different plugs, and with diverse size, from those used for micro-CT analysis, even though drilled from the same tuff block. However, it can be useful to quickly discuss the threshold sensitivity^34^ for the segmentation of the acquired scans. In fact, image segmentation is a crucial step for digital rock physics, but it is widely affected by the user/algorithm-selected threshold values. We explored how the porosity changes with thresholding variations from the Otsu value. Otsu thresholding, in fact, proved to be among the best possible approaches to capture textural features of our rocks without further geological verifications (for details on geological driven workflows for rock segmentation please refer to Balcewicz et al.^35^). Our data shows that HR and VHR scans provide porosity values little affected by user/algorithm-selected parameters, while LR scan is very sensitive to threshold variations (Fig. 2b).

In summary, the multiscale study of our tuff core shows that the investigation of small portions of tuff rocks with high resolution can allow more accurate estimates of their petrophysical properties. However, great attention must be paid to the risk of non-representativeness when very small rock volumes are explored due to the high heterogeneity that usually characterize volcanic rocks^3,36^. Moreover, high resolutions scans may require excessively long scan times or hard X-rays, especially when only large rock samples are available^15^. In order to deal with these limitations, we examined the effectiveness of deep learning-based super-resolution approaches on this type of digital rocks.

### Super-resolution imaging

Deep learning-based super-resolution methods can allow to improve the quality of an image learning mapping functions from low resolution to high resolution images. Evaluating their efficacy on 3D images of rocks can critically improve digital rock physics workflows (e.g., to achieve large fields of view with high resolution or enhance fast 4D low quality scans). Although pioneering efforts have been made to apply these methods to digital rocks, they mainly focused on sedimentary rocks and employed LR images synthetically downsampled from HR images^20,25^. Janssens et al.^18^ recently showed that synthetically downsampled LR images are often able to retain microstructure complexities, largely compromised in real LR scans by imaging artefacts.

We tested six different robust convolutional neural network (CNN) and generative adversarial network (GAN)-based super-resolution approaches (U-Net; SR-ResNet; EDSR; WDSR-a; WDSR-b, pix2pix; see “Materials and methods” section for details on the networks and their training) using corresponding paired volumes of our real LR scan (input data) and HR scan (ground truth data) of microporous volcanic tuff (Fig. 1d), aimed at finding effective and quick methods to optimize the investigation of these rocks. HR scan, in fact, proved to adequately capture transport properties with a more sustainable scaling factor (4× vs. 10×) and scan time (3.2 vs. 14 s per projection) than VHR scan. It is worth noting that LR crop synthetically downsampled (with cubic interpolation) from our HR image shows porosity values closer (0.55) to the HR crop (0.49) than those of the real LR crop (0.74), consistently with the findings of Janssens et al.^18^.

We first employed 2D CNN architectures distinguishable in two groups: U-Net and ResNet-based (SR-ResNet, EDSR, WDSR-a, WDSR-b) networks. U-Net network leads to significant enhancement of the image quality (Figs. 3, 4), corroborated by excellent pixelwise accuracy of the validation data. In fact, super-resolved validation images, when compared to their ground truth (corresponding HR images), show PSNR (peak signal to noise ratio) of 28.6 dB, MSE (mean squared error) of 0.0014 and SSIM (structure similarity index) of 0.80 (Table 1). Consequently, the total porosity calculated from their segmentation (Fig. 4) is consistent with laboratory data and weakly sensitive to threshold variations, in contrast with their equivalent LR images (both LR scan and LR scan upsampled to HR size with cubic interpolation) which provide an overpredicted total porosity (Fig. 2, Table 1). Conversely, all the employed ResNet-based networks seem inadequate to improve the quality of the images and to simplify their processing. Indeed, the application of these trained models to the validation images mainly results in too smoothed/blurred images, unable to resolve the small pores and the fine matrix (Fig. 3). This is supported by low image quality metrics: PSNR of 27.2 dB, MSE of 0.0019 and SSIM of 0.70–0.71 (Table 1). Accordingly, their segmentation is still hindered by the same difficulties described above for the LR images (see “Multiscale imaging” section) and leads to porosity values even more divergent from laboratory data than the equivalent original LR input images (Table 1). These findings are possibly due to the less complex architectures than U-Net, attested by the lower number of trainable parameters (one order of magnitude less than U-Net) and shorter training times for the same number of epochs (Table 1). Moreover, unlike U-Net, LR images are directly upsampled within these network. Thus, U-Net requires a preliminary LR image upsampling to HR size that augment data, while ResNet-based networks need to discard some HR images (depending on the scaling factor) in order to work with corresponding pairs of input and ground truth images (Table 1).

**Table 1:** Training details and image quality metrics for the trained 2D and 3D CNNs and GANs.

Model	Network parameters	Patches (training + validation)	Training epochs	Training time (h:m:s)	MSE	PSNR (dB)	SSIM	Porosity (Otsu)
LR cubic	–	–	–	–	0.0073	21.36	0.64	0.68
U-Net 2D	3.10 × 10^7^	24,300	100	03:32:35^a^	0.0014	28.58	0.80	0.47
SR-ResNet 2D	1.53 × 10^6^	3888	100	00:28:00^a^	0.0019	27.21	0.71	0.73
EDSR 2D	1.52 × 10^6^	3888	100	00:16:28^a^	0.0019	27.23	0.71	0.73
WDSR-A 2D	1.19 × 10^6^	3888	100	00:14:46^a^	0.0019	27.22	0.70	0.73
WDSR-B 2D	1.29 × 10^6^	3888	100	00:27:01^a^	0.0019	27.22	0.70	0.73
pix2pix 2D	G: 4.18 × 10^7^, D: 6.96 × 10^6^	24,300	100	44:07:44^a^	0.0013	28.82	0.82	0.48
U-Net 3D	9.03 × 10^7^	1400	100	17:55:12^a^	0.0019	27.31	0.70	0.65
pix2pix 3D	G: 1.67 × 10^8^, D: 2.78 × 10^7^	288	100	04:08:33^a^	0.0020	26.91	0.68	0.52
CycleGAN 2D	G: 1.84 × 10^7^, D: 6.96 × 10^6^	24,300 (unpaired)	25	37:05:08^b^	–	–	–	–
CycleGAN 3D	G: 5.54 × 10^7^, D: 2.78 × 10^7^	288 (unpaired)	25	12:04:35^b^	–	–	–	–

Image quality metrics (respect to HR images), together with porosity values, were obtained from validations slices. Network parameters for GANs (pix2pix and CycleGAN) are reported for generators (G) discriminators (D). Training time refers to two different GPUs: NVIDIA GeForce RTX 3070 (a) and NVIDIA GeForce RTX 3090 Ti (b). CycleGAN was trained on unpaired images, which do not allow to estimate image quality metrics; validation data looks promising from a visual inspection, but still not recommendable for estimating transport properties (see text for details).

The resulting best 2D CNN, U-Net, was then tested as GAN generator implementing a pix2pix network; these two models were explored both in 2D and 3D. The trained 2D pix2pix network provides substantial improvements of the image quality and pixelwise accuracy of validation data, even slightly better than 2D U-Net, although longer training times are required for the same number of epochs: PSNR of 28.8 dB, MSE of 0.0013 and SSIM of 0.82 (Table 1, Figs. 3, 4). Their segmentation is efficient (Fig. 4), resulting in porosity values consistent with laboratory data (Table 1). Particularly, 2D pix2pix is able to better detect high-frequency structures than 2D U-Net as expected (see “Materials and methods” section). It allows to segment small-scale textural features (such as microcracks in crystals), however the slight blurring/smoothing effects of 2D U-Net (acting as denoising) is sometimes useful as well. 3D U-Net and pix2pix networks, instead, produce worse results, resulting in bad images (Fig. 4), image quality metrics (PSNR of 26.9–27.3 dB, MSE of 0.0019–0.0020 and SSIM of 0.68–0.70; Table 1) and porosity values (due to the consequent difficulty in properly resolving and segmenting small pores and matrix grains) for validation data (Table 1). It is possibly due to the resulting lower number of training patches than their 2D counterpart (Table 1).

**Figure 5:**
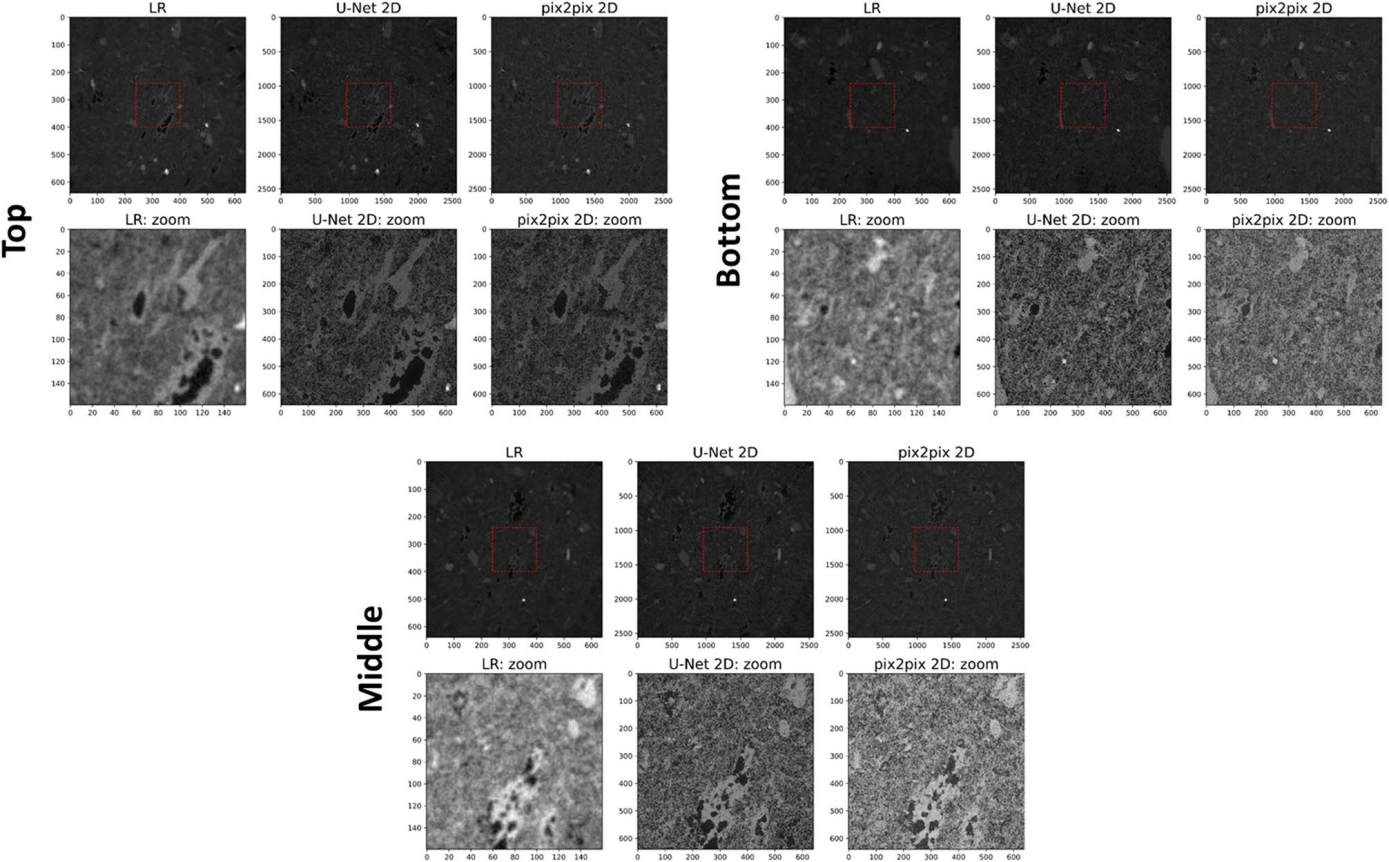
Super-resolved large tuff core: best models. Super-resolved images (2560^3^ px) obtained applying the best trained networks, 2D pix2pix and U-Net trained on paired data, to a large unseen LR image (central 640^3^ px; i.e., dominantly external to the training/validation dataset). LR images (input) are also shown for comparison. Slices from top, middle and bottom of the whole 3D images are presented, together with a zoom in their central portion in order to better detect the reconstructed microstructures.

**Figure 6:**
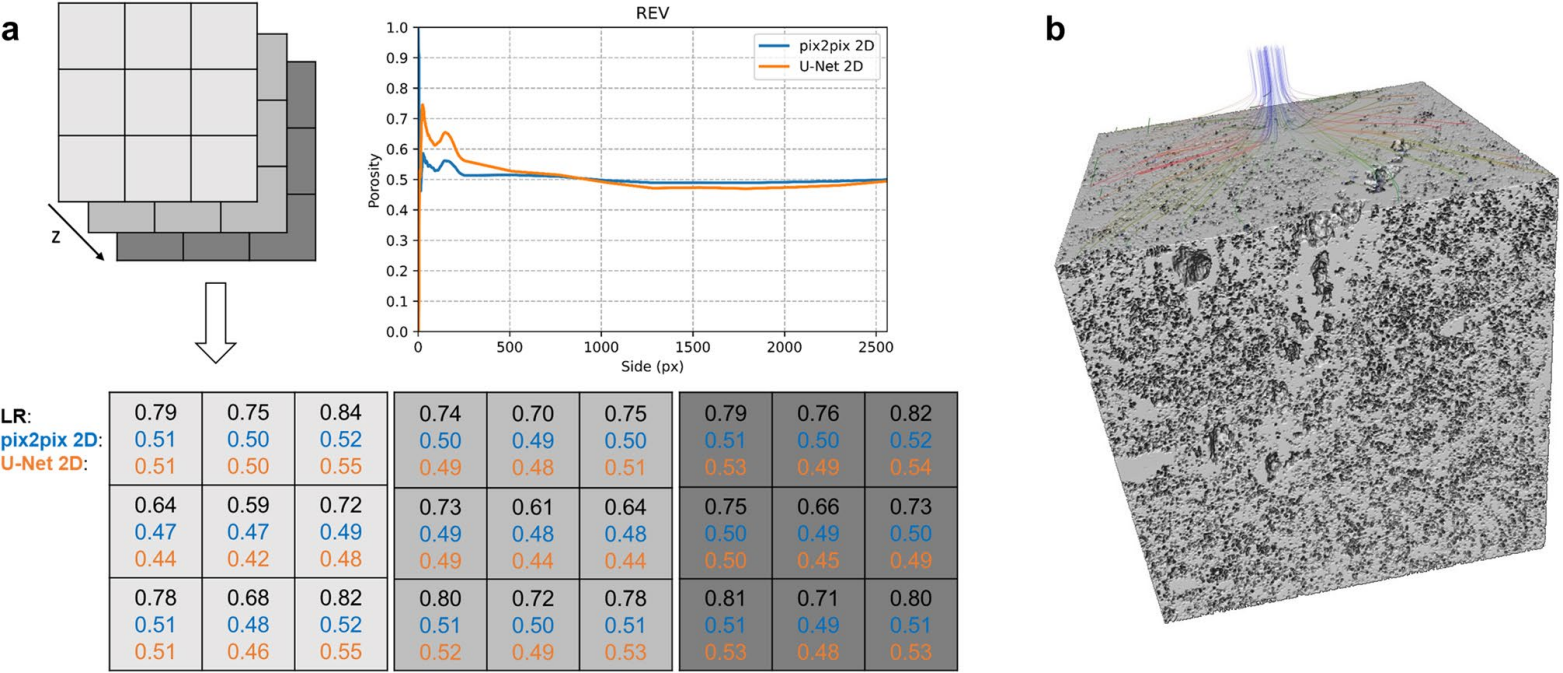
Petrophysical features of the super-resolved large tuff core. Petrophysical measurements performed on the super-resolved images (2560^3^ px) obtained applying the best trained networks, 2D pix2pix and U-Net trained on paired data, to a large LR image (central 640^3^ px; see Fig. 5). (**a**) Porosity values estimated using the box-counting method to calculate the minimum Representative Elementary Volume, REV (top), as well as dividing the super-resolved images in adjacent representative (i.e., larger than the minimum REV) subvolumes to detect potential heterogeneities (bottom). (**b**) Example of 3D super-resolved (2D U-Net) and segmented image employed for intrinsic permeability simulations (central 900^3^ px, the maximum volume exploitable for our computational system).

In order to test the overall effectiveness of our best trained models, 2D U-Net and pix2pix, we applied them to a large unseen (i.e., dominantly external to the training/validation dataset) LR image volume and estimated their transport properties using the described image analysis workflow. Applying the models on the central 640^3^ px of the LR image we obtained a super-resolved 3D image of 2560^3^ px with a substantial quality enhancement (Fig. 5), that allow to image a large field of view with high resolution. The estimated petrophysical properties are almost consistent with laboratory data and weakly sensitive to threshold variations (Fig. 2b). Particularly, while the original LR image shows an apparent highly porous, permeable and heterogeneous microstructure, super-resolved images have porosity, permeability and structural uniformity coherent with laboratory data and macroscopic features (Figs. 2b, 6).

**Figure 7:**
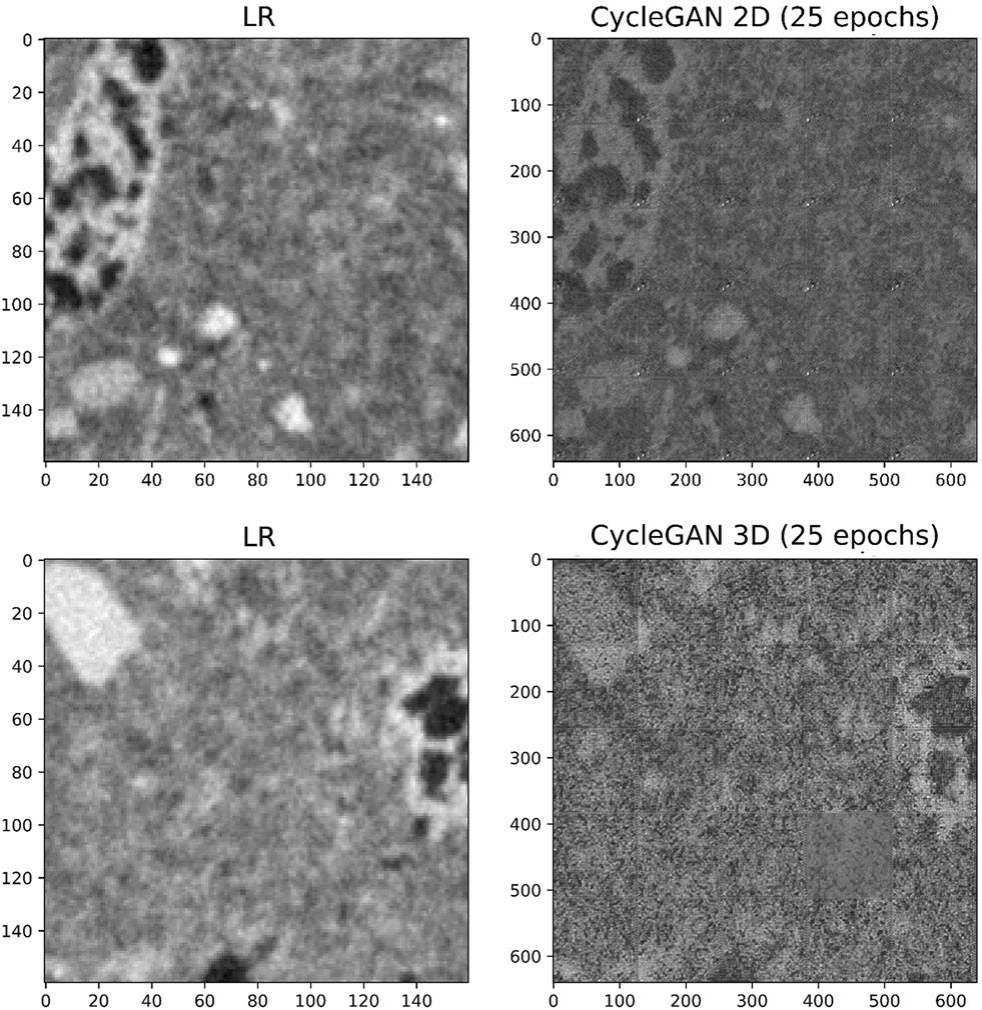
Super-resolved validation images: 2D and 3D networks trained on unpaired data. CycleGAN was trained both in 2D and 3D on unpaired data (i.e., using non-corresponding LR and HR images). LR (input) images are also shown for comparison. Correspondent training details are reported in Table 1. A relatively limited training of 25 epochs (455,625 and 5750 training steps in 2D and 3D) was possible in reasonable times due to the high computational costs (see also Table 1).

Finally, we also explored CycleGAN, a cycle‐consistent adversarial network specifically developed to learn the mapping between unpaired (non-corresponding) training images (e.g., from LR to HR data), trained both in 2D and 3D for this challenging task (see “Materials and methods” section for details on network and training). Here we show data obtained with the relatively limited trainings attainable in reasonable times by our dedicated GPUs (25 epochs: 455,625 and 5750 training steps in 2D and 3D; Table 1), in order to explore the feasibility of super-resolving our low resolution scan in the absence of paired LR-HR data, not always readily available. Our results appear promising (especially in 2D), although moderately affected by inconstancy throughout the training, stitching artefacts and limitations in recovering edges and matrix/crystal uniformity (Fig. 7). This makes them still not recommendable for estimating transport properties because of difficulties in segmenting images with full geological validity. This is possibly due to limited trainings (aggravated in 3D by the fewer training data) when compared to the complex microstructure of these rocks, containing small (not easily resolvable) and heterogeneous pores and matrix grains. In fact, pioneering efforts made using similar networks on real sedimentary digital rocks led to suitable results^22,24^.

## Conclusions

Digital rock physics workflows are powerful tools to explore rock microstructure and physical properties. However, very few efforts have been done so far to investigate microporous volcanic rocks despite their wide range of applications, possibly due to their challenging microstructures. Here we explored methods to optimize the 3D non-destructive characterization of these rocks, focusing on the Campanian Ignimbrite tuff, the dominant product of the largest Quaternary eruption in Europe.

A multiscale imaging of a core with progressively decreasing pixel sizes (from 16 to 1.75 μm) and fields of view, and increasing exposure time, was performed. We found that more accurate estimates of the petrophysical properties can be achieved examining small volumes of tuffs with high (≤ 4 μm/px) resolution, rather than large fields of view with smaller resolutions. The main limitations are that representative volumes must be guaranteed and high resolutions scans may require excessively long scan times or hard X-rays (unless small samples are used). Therefore, we explored the effectiveness of deep learning-based super-resolution approaches, implementing and comparing many robust 2D and 3D, convolutional neural networks and generative adversarial networks trained on paired LR-HR data, to artificially image large fields of view of our microporous volcanic rock with high resolutions. 2D U-Net and pi2pix networks guaranteed excellent image quality enhancements (compared to ground truth: PSNR of 28.6–28.8 dB, MSE of 0.0013–0.0014 and SSIM of 0.80–0.82) and petrophysical properties almost consistent with laboratory measurement, in contrast with ResNet-based (SR-ResNet, EDSR, WDSR-a, WDSR-b) and 3D networks. Finally, 2D/3D CycleGAN, a cycle‐consistent adversarial network, was also employed to test the feasibility of super-resolving our scan using unpaired LR-HR training data. It led to promising results, although limited by high computational costs, offering positive prospective to achieve this demanding task on complex volcanic rocks.

It is, to our knowledge, the first time that deep learning-based super-resolution methods are tested on non-sedimentary rock images and one of the few studies that apply these approaches to real LR and HR images (i.e., not synthetically downsampled but truly acquired and registered).

Although attention must be paid to extend our results to all the types of microporous volcanic rocks, this study represents the first effort to systematically explore these “unconventional” digital rocks through digital rock physics, as well as offers a framework to deal with similar microporous structures and innovative imaging challenges (e.g., high-resolution imaging of large samples or materials to be preserved, 4D imaging with large experimental apparatus and/or high temporal resolution). Moreover, Campanian Ignimbrite tuff is thought to be one the most widespread subsurface rock at the Campi Flegrei caldera^37,38^, currently in a state of unrest since 2005^28,29^. Our findings will be employed in future studies to optimize the 3D investigation of core samples from geothermal drills in this area and time-resolved (4D) imaging during in-situ and/or ex-situ experiments to better constrain the ongoing dynamics at this large active caldera.

## Materials and methods

### Materials and laboratory estimation of hydraulic properties

We investigated the Campanian Ignimbrite (CI) tuff, the main product of the homonymous volcanic eruption occurred from the Campi Flegrei caldera (Italy) 39 ka ago^39^, considered the largest Quaternary eruption in Europe. This eruption produced a minor basal fallout, dispersed to the E–NE, overlain by a dominant, sub-radial, stratified pyroclastic density current (PDC) deposit. The Welded Gray Ignimbrite (WGI) is the most widely distributed unit and constitutes most of the CI thickness^26^. The CI tuff originated from the rapid deposition and lithification of hot PDCs with a volume of about 453–606 km^3^, covering an area of more than 6000 km^2^^26^ (Fig. 1a). This tuff is the most widespread among the caldera-filling deposits^37,38^ extended up to a depth of ~ 4 km^5^. It was thus investigated for numerous (mainly volcanological, geothermal, civil and material engineering) applications^3–5,26,29^.

We studied a WGI tuff block collected from an open quarry for building materials close to the city of Caserta (Fig. 1a), consisting of black scoriae embedded in an ashy matrix with subordinate lithics and crystals (mainly feldspars and pyroxene). It shows a complex microstructure in which the pores are distributed in a very fine, lithified, heterogeneous matrix (Fig. 1b). We estimated some its hydraulic properties, i.e., hydraulic conductivity, intrinsic permeability and total porosity, by using laboratory tests and empirical formulas. Particularly, hydraulic conductivity measurements were obtained by testing 2 rock samples at the Laboratory of Geological and Geotechnical Engineering of the Department of Earth, Environmental and Resources Sciences of the University of Naples Federico II. An irregular block of CI tuff was shaped using a coring machine (MATEST, Italy, A140-01 model), an electric saw (Husqvarna, Italy, TS 230 F model) and a polisher machine (Buehler, Germany, AutoMet Grinder-Polishers model) to obtain 54 mm diameter and 103 mm height two polished cylindrical specimens. The cylindrical rock specimens were prepared according to the standard (ASTM D4525–90). Hydraulic conductivity measurements were performed on saturated rock samples, after immersing the specimens in distilled water for 4 days. For two rock specimens, 12 and 18 hydraulic tests of hydraulic conductivity were performed. Hydraulic conductivity tests were carried out according to the ASTM standards (ASTM D2434-68, ASTM D5084-16a) by a triaxial apparatus with Hoek cell (MATEST, Italy, A137 model). In Allocca et al.^1^ further details of laboratory instruments and procedure used for measurements of hydraulic conductivity are reported. Hydraulic conductivity, *K* (m/s), was estimated by using Darcy’s law in the steady-state flow condition, and subsequently converted in intrinsic permeability, *k*_*i*_ (mD), by following equations:

1 $$K=\frac{q}{A\times i},$$

2 $$ k_{i} = \frac{{K \times \mu_{w} }}{{\rho_{w} \times g}}, $$

where *q* is the volumetric flow-rate of water (m^3^/s), *A* is the cross-sectional area of the cylindrical specimen (m^2^), *i* is the hydraulic gradient (dimensionless), *μ*_*w*_ and *ρ*_*w*_ are water viscosity (N × s/m^2^) and density (kg/m^3^) at ambient conditions respectively, and *g* (m/s^2^) is the gravitational constant. Finally, total porosity, *φ* (dimensionless) was empirically estimated using dry bulk density,* ρ* (kg/m^3^) previously determined in laboratory, and dense rock equivalent density (*ρ*_*DRE*_ = 2.607 ± 31 kg/m^3^^26^) by following empirical formula^40^:

3 $$ \varphi = \frac{{\rho_{DRE} - \rho }}{{\rho_{DRE} }}. $$

### Multiscale X-ray imaging

X-ray microtomography investigations were performed on a cylindrical core with diameter of about 20 mm and a height of 40 mm from the same tuff block (Fig. 1c). We examined the sample at multiple scales, progressively decreasing pixel sizes and fields of view, and increasing exposure time. We acquired three 3D images (of about 1000^3^ px): a low resolution (LR) scan with a pixel size of about 16 μm, a high resolution (HR) scan with a pixel size of 4 μm and a very high resolution (VHR) scan with a pixel size of 1.75 μm (Fig. 1c). The scans were acquired at the micro-CT laboratory of the Istituto Nazionale di Geofisica e Vulcanologia—Osservatorio Vesuviano, equipped with a ZEISS Xradia Versa 410 micro-CT. X-ray imaging was performed in absorption mode acquiring 2D radiographs (projections) over a total angular scan of 360°, reconstructed with a filtered back-projection algorithm using the XRM Reconstructor software. LR scan was scanned at 80 kV and 7 W, using an optical magnification of 0.4× and collecting 4001 projections with a scan time of 3 s per projection. HR scan was scanned at 100 kV and 9 W, using an optical magnification of 4× and collecting 4001 projections with a scan time of 3.2 s per projection. VHR scan was scanned at 100 kV and 9 W, using an optical magnification of 10× and collecting 3201 projections with a scan time of 14 s per projection. For HR and VHR scans, a low-energy (LE6) filter was used to minimize beam hardening.

### Super-resolution

Super-resolution methods based on convolutional neural networks and generative adversarial networks are proving to be particularly efficient to enhance the quality of low resolution images in numerous applications. Here, we tested the effectiveness of these approaches on images of tuff rock with challenging microstructures. We used real LR and HR scans (see “Multiscale X-ray imaging” section), with a scaling factor of 4×, opportunely preprocessed, and several 2D-3D, CNNs and GANs-based networks.

#### CNNs and GANs-based super-resolution

**Figure 8:**
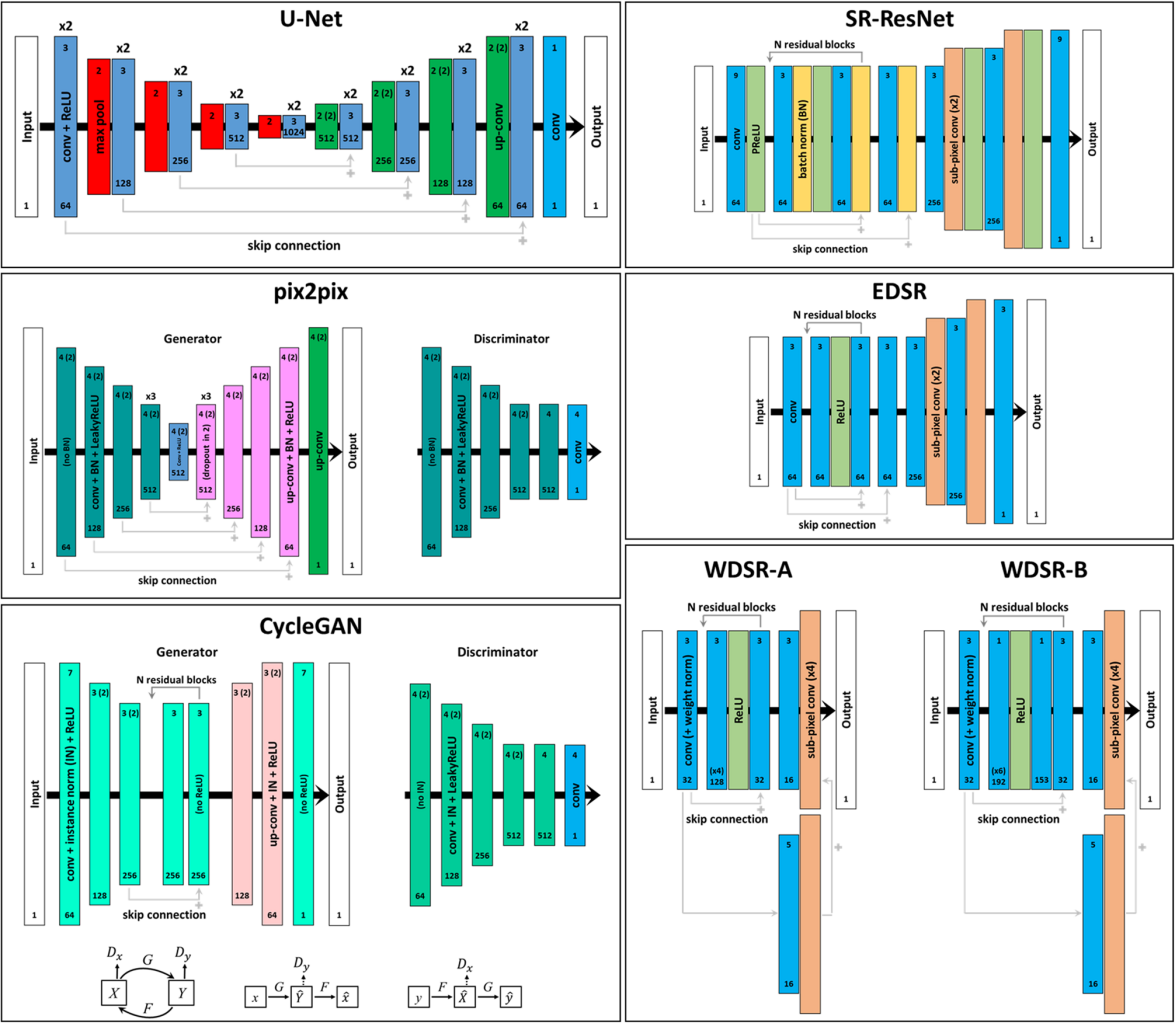
Super-resolution networks. Convolutional neural networks (CNNs: U-Net, SR-ResNet, EDSR, WDSR-a, WDSR-b) and generative adversarial networks (GANs: pix2pix, CycleGAN) employed for super-resolution imaging. Blocks of the same color represent the same type of layer(s); if they are repeated for several times, it is reported above the blocks (e.g., 2×). For (2D or 3D) convolutional layers, the number of filters is provided at the bottom and the kernel size (together with the strides, in the bracket, when different from 1) at the top. Bottom light-gray arrows show skip connections (concatenation for U-Net, pix2pix, CycleGAN, addition for SR-ResNet, EDSR, WDSR-a, WDSR-b), whereas top dark-gray arrows indicate residual blocks for ResNet-based networks (SR-ResNet, EDSR, WDSR-a, WDSR-b) and CycleGAN.

We trained seven different networks which have shown very effective and quick results in numerous scientific and computer vision fields and challenges (U-Net, SR-ResNet, EDSR, WDSR-a, WDSR-b, pix2pix, CycleGAN; Fig. 8). We mainly used corresponding pairs of LR (input data) and HR (ground truth data) images, progressively focusing on the networks raveled more efficient for the microstructure of our tuff after apposite parametric studies. 2D CNNs (U-Net and ResNet-based CNNs: SR-ResNet, EDSR, WDSR) were initially employed. The resulting best model (U-Net) was then used both in 2D and 3D, and as generator of a GAN (pix2pix). Finally, we tested a cycle‐consistent adversarial network (CycleGAN) specifically developed to learn the mapping between unpaired (non-corresponding) training images (e.g., from LR to HR data) both in 2D and 3D.

Deep convolutional neural networks, CNNs, are a class of neural networks that have convolutional layers as building blocks. They convolve an input (a tensor with shape: number of inputs, input height, input width, input channels, in 2D; initially batches of input images) with a set of convolutional filters (with shape: number of filters, kernel height, kernel width, in 2D) giving a feature map as output; thus number and shape of convolutional filters crucially impact on the total resulting trainable parameters. CNNs typically combine convolutional layers with activation, batch normalization, downsampling and upsampling layers, and minimize specific loss functions by an optimizer to solve regression (e.g., super-resolution) or classification (e.g., semantic segmentation) tasks^20,41^. For super-resolution tasks, CNNs learn the LR to HR scan mapping. In this study, we first adopted different kinds of CNN architectures, distinguishable in two groups: U-Net and ResNet-based networks. The effectiveness of both is enhanced by the use of skip connections that, adding together outputs from shallow and deep layers, allow to preserve shallow features and optimize the training (avoiding vanishing gradient and degradation problems). U-Net architecture, originally developed for semantic segmentation, has been used for numerous tasks^42^. It works with input and output images with the same shape, it is thus required a preliminary upsampling of the LR scans (to HR size) for super-resolution. U-Net uses a contracting path to capture the context through downsampling steps (i.e., convolution plus max pooling layers) and a symmetric expanding path (hence U-shape; Fig. 8) for a precise localization through upsampling (up-convolution) and concatenation with corresponding feature maps from the contracting path (skip connection). On the other hand, SR-ResNet is a super-resolution network, directly built on ResNet backbone^43^ (Fig. 8). ResNet was originally developed to solve image recognition tasks using residual blocks with skip connections to train very deep networks^44^. EDSR optimizes the SR-ResNet architecture, mainly removing batch normalization and changing activation layers (from PReLU, Parametric Rectified Linear Unit, to ReLU) to increase the performance^45^ (Fig. 8). Finally, WDSR, starting from EDSR architecture, tests the use of wider features before the activation function with same parameters and computational budgets, and introduce weight normalization (rather than batch normalization or no normalization). Based on their width two different types are proposed: WDSR-a for wide 2×–4× channels, WDSR-b for even wider 6×–9× channels^46^ (Fig. 8). These ResNet-based CNNs works directly on LR images and data are upsampled within the network through pixel shuffling (sub-pixel convolution). Further information for digital rocks are provided by Wang et al.^47^.

In contrast to CNNs, generative adversarial networks, GANs, are composed of two networks: a generator model learns to generate fake images, a discriminator model learns to classify images as real (training images) or fake (generated images). They are simultaneously trained in an adversarial process, during which the generator tries to create progressively more realistic images to fool the discriminator, which in turn tries to better identify fake images. The discriminator is directly updated, the generator is updated through the discriminator^48^. GANs usually learn a mapping from random noise vectors to output data (in this case images) but can be conditioned on some extra information, such as input LR images; in that case they are called conditional GANs (c-GANs^49^). Thus, using an efficient CNN network as generator and an appropriate image classification network as discriminator, c-GANs can be efficient for image-to-image mappings. Here, we employed pix2pix^50^, a c-GAN with a generator based on a U-Net architecture (revealed the best CNN for our rock). It was developed to be a general-purpose solution to many image-to-image translation tasks^50^ and has been recently employed also for multimodal imaging of digital rocks^51,52^. The discriminator network, called PatchGAN, is a multi-layer CNN classifier mainly restricted to model high-frequency structures in local image (70 × 70 px) subpatches, on which it classifies an image as real or fake (Fig. 8). In fact, CNNs (commonly based on mean absolute error or mean squared error loss) can blur/smooth high-frequency structures^43,50^, resulting in advantages for segmentation (thus acting as denoising) or in the loss of important small-scale textural information depending on the investigated microstructures and images^20^. pix2pix loss combines a c-GAN loss with a L1 (mean absolute error) loss between generated and expected images, that encourages the generation of image similar to the ground truth.

Finally, CycleGAN^53^ is an adversarial network specifically developed to learn mapping functions between two domains (*X* and *Y*) in the absence of paired images. To address this under-constrained problem, it uses two generators (*G*: *X → Y* and *F*: *Y → X*), based on a modified ResNet backbone, and two associated adversarial discriminators (*D*_*y*_ and *D*_*x*_), two 70 × 70 PatchGANs, adopting instance normalization (Fig. 8). In addition to the adversarial losses, two cycle consistency losses are introduced to regularize these mappings so that: *x → G(x) → F(G(x))* *≈* *x* and *y → F(y) → G(F(x))* *≈* *y* (Fig. 8). Identity mapping losses are also employed to regularize the generator to be near an identity mapping when images from the target domain are provided as inputs. CycleGAN was built to be a general solution for image-to-image translation^53^ and has been recently applied to super-resolve digital rocks^22^.

#### Data preparation and training

We applied super-resolution methods to our LR scan (input data) and HR scan (ground truth data), which adequately captures transport properties (see “Multiscale imaging” section) with a more sustainable scaling factor (4× vs. 10×) and scan time (3.2 vs. 14 s per projection) than VHR scan. Particularly, we used LR and HR crops opportunely registered and cropped. 3D registration was refined with Thermo Scientific PerGeos Software (based on Avizo Software; Thermo Fisher Scientific, Waltham, MA, USA - www.thermofisher.com/pergeos) maximizing the normalized mutual information between the two scans^54^, thus resulting in a slight translation, rotation and scaling of LR scan with a final pixel size of 16 μm. A crop of 640 × 640 × 972 px (i.e., the largest inscribable parallelepiped in our cylindrical reconstructed digital rock compatible with our networks) was extracted from the HR scan, and a corresponding volume of 160 × 160 × 243 px from the LR scan (Fig. 1d), to obtain training and validation paired data. For U-Net and pix2pix, LR crop was upsampled to HR size with cubic interpolation. A larger crop of 640^3^ px was extracted from the LR scan to test the best models.

LR and HR crops were preprocessed to optimize the training of the networks after parametric tests. For 2D networks, 25% of the slices were randomly selected for validation; the images were subdivided in patches of 128^2^ px for 2D U-Net and pix2pix, and of 40^2^ and 160^2^ px (for LR and corresponding HR images, respectively) for ResNet-based networks (revealed adequate sizes to capture tuff microstructures). 2D best models, U-Net and pix2pix, were also trained in 3D preparing subvolumes of 80^3^ and 128^3^ px respectively (also constrained by our computational availability) with an overlap of 25% to augment data; 20% of the subvolumes was used for validation and the last 80/128 slices were reserved to evaluate the general result of the models.

For 2D and 3D CNNs (U-Net and ResNet-based networks), grayscale values were scaled from 0 to 1 and a sigmoid activation layer was used at the end of the networks. They were trained for 100 epochs using mean squared error loss (which provided better results than mean absolute error loss) and Adam optimizer with an initial learning rate of 10^–3^ and an exponential decay (decay rate of 0.0625). In detail, for U-Net, batches of 32 and 1 images were used for 2D and 3D models respectively, shuffling the training data before each epoch; the effect of BatchNormalization and Dropout was also investigated without enhancements. For ResNet-based networks, results for 16 residual blocks using shuffled batches of 16 images, and for filter expansion factors of 4× and 6× for WDSR-a and WDSR-b respectively, are shown; however changing these parameters no significant improvements were achieved. For pix2pix, we followed the suggestions proposed by its authors as it was developed as a general-purpose solution to many different image-to-image translation tasks^50^. Grayscale values were scaled from − 1 to 1 and a tanh activation layer was used at the end of the generator. The model was trained for 100 epochs using a batch size of 1, randomly selecting the images. The number of epochs to train CNNs and GANs was selected in order to broadly stabilize the metrics for training and validation datasets. The trainings were performed using Tensorflow/Keras (Tensorflow 2.5.0, Python 3) on a NVIDIA GeForce RTX 3070 GPU.

The results were then evaluated in term of image quality metrics and transport properties (see “Image analysis and flow simulations” section), and visual checks as well. Image quality (respect to HR images) was assessed estimating mean squared error (MSE), peak signal to noise ratio (PSNR) and structure similarity index (SSIM^55^).

Finally, we also used unpaired data to train 2D and 3D CycleGANs. HR data was combined with a stack of 2D images from different FoVs, and a 3D subvolume, independently extracted from the central 640^3^ px of the LR image and upsampled with cubic interpolation. These datasets were preprocessed similarly to (2D and 3D) pix2pix. We trained CycleGAN following the suggestions proposed by its authors, who developed a general solution for image-to-image translation tasks^53^, and randomly selecting the images. A relatively limited training of 25 epochs (455,625 and 5750 training steps in 2D and 3D) was possible in reasonable times (Table 1) due to the high computational costs, although a better performing GPU (NVIDIA GeForce RTX 3090 Ti) was employed for this network. The absence of paired images, did not allow the use of traditional image quality metrics to evaluate the results.

### Image analysis and flow simulations

The obtained digital rocks were segmented and used to estimate transport properties. We defined a very simple and traditional workflow in order to evaluate the efficiency of our imaging of tuff microstructures without further complications due to filtering, segmentation and simulation approaches. Therefore, we fist segmented the images using Otsu’s method^56^, which automatically estimate a grayscale threshold for a binary segmentation (i.e., each voxel is labeled as pore or matrix), with scikit-image library in Python. The results were visually checked and the effect of threshold value on segmentation (i.e., threshold sensitivity^34^) is also discussed. The segmented images we then used to estimate porosity and perform permeability simulations. Particularly, single-phase fluid flow was directly simulated on the segmented images. This approach allows to estimate permeability (compatible with our laboratory measurements) and velocity field distribution, solving Stokes equations and using Darcy’s law. We employed a parallel lattice Boltzmann solver available in the PerGeos software.

## Data Availability

Supporting data and codes are available at: https://figshare.com/articles/online_resource/3dSRCT/20449188.

## References

[1] Allocca V, Colantuono P, Colella A, Piacentini SM, Piscopo V (2022). Hydraulic properties of ignimbrites: Matrix and fracture permeabilities in two pyroclastic flow deposits from Cimino-Vico volcanoes (Italy). Bull. Eng. Geol. Environ..

[2] Bonamente E, Aquino A, Nicolini A, Cotana F (2016). Experimental analysis and process modeling of carbon dioxide removal using tuff. Sustainability.

[3] Heap MJ, Violay MES (2021). The mechanical behaviour and failure modes of volcanic rocks: A review. Bull. Volcanol..

[4] Heiken, G. *Tuffs-Their Properties, Uses, Hydrology and Resources. Geological Society of America (GSA) Special Paper*, Vol. 408. 10.1130/SPE408 (2006).

[5] Rosi, M. & Sbrana, A. *The Phlegrean Fields. CNR Quaderni de La Ricerca Scientifica* 114 (1987).

[6] Rowley P, Benson PM, Bean CJ (2021). Deformation-controlled long-period seismicity in low-cohesion volcanic sediments. Nat. Geosci..

[7] Wang J, Jung W, Li Y, Ghassemi A (2016). Geomechanical characterization on Newberry tuff. Geothermics.

[8] Zou C (2013). Volcanic Reservoirs in Petroleum Exploration.

[9] Fisher RV, Schmincke H-U (1984). Pyroclastic Rocks.

[10] Brown RJ, Andrews GDM, Sigurdsson H, Houghton B, McNutt SR, Rymer H, Stix J (2015). Deposits of pyroclastic density currents. The Encyclopedia of Volcanoes.

[11] Andrä H (2013). Digital rock physics benchmarks part I: Imaging and segmentation. Comput. Geosci..

[12] Andrä H (2013). Digital rock physics benchmarks part II: Computing effective properties. Comput. Geosci..

[13] Blunt MJ (2013). Pore-scale imaging and modelling. Adv. Water Resour..

[14] Bultreys T, De Boever W, Cnudde V (2016). Imaging and image-based fluid transport modeling at the pore scale in geological materials: A practical introduction to the current state-of-the-art. Earth Sci. Rev..

[15] Withers PJ (2021). X-ray computed tomography. Nat. Rev. Methods Primers.

[16] Alyafei N, Raeini AQ, Paluszny A, Blunt MJ (2015). A sensitivity study of the effect of image resolution on predicted petrophysical properties. Transp. Porous Media.

[17] Arns CH (2005). Pore-scale characterization of carbonates using X-ray microtomography. Soc. Pet. Eng. J..

[18] Janssens N, Huysmans M, Rudy S (2020). Computed tomography 3D super-resolution with generative adversarial neural networks: Implications on unsaturated and two-phase fluid flow. Materials.

[19] Shah SM, Gray F, Crawshaw JP, Boek ES (2016). Micro-computed tomography pore-scale study of flow in porous media: Effect of voxel resolution. Adv. Water Resour..

[20] Wang YD, Blunt MJ, Armstrong RT, Mostaghimi P (2021). Deep learning in pore scale imaging and modeling. Earth Sci. Rev..

[21] Ahuja VR (2022). Siamese-SR: A siamese super-resolution model for boosting resolution of digital rock images for improved petrophysical property estimation. IEEE Trans. Image Process..

[22] Chen H (2020). Super-resolution of real-world rock microcomputed tomography images using cycle-consistent generative adversarial networks. Phys. Rev. E.

[23] Karimpouli S, Kadyrov R (2022). Multistep super resolution double-U-net (SRDUN) for enhancing the resolution of Bereasandstone images. J. Pet. Sci. Eng..

[24] Niu Y, Jackson SJ, Alqahtani N, Mostaghimi P, Armstrong RT (2022). Paired and unpaired deep learning methods for physically accurate super-resolution carbonate rock images. Transp. Porous Media.

[25] Rabbani A (2021). Review of data science trends and issues in porous media research with a focus on image-based techniques. Water Resour. Res..

[26] Silleni A, Giordano G, Isaia R, Ort MH (2020). Magnitude of the 39.8 ka Campanian Ignimbrite Eruption, Italy: Method, uncertainties and errors. Front. Earth Sci..

[27] Buono G (2020). Dynamics of degassing in evolved alkaline magmas: Petrological, experimental and theoretical insights. Earth Sci. Rev..

[28] Buono G (2022). New insights into the recent magma dynamics under Campi Flegrei caldera (Italy) from petrological and geochemical evidence. J. Geophys. Res. Solid Earth.

[29] Pappalardo L, Buono G, Masotta M, Beier C, Mollo S (2021). Insights into processes and timescales of magma storage and ascent from textural and geochemical investigations: Case studies from high-risk Neapolitan Volcanoes (Italy). Crustal Magmatic System Evolution.

[30] Baker DR (2012). An introduction to the application of X-ray microtomography to the three-dimensional study of igneous rocks. Lithos.

[31] Buono G, Pappalardo L, Petrosino P (2020). Magma storage and ascent during the largest eruption of Somma-Vesuvius volcano: Pomici di Base (22 ka) Plinian event. Boll. Geofis. Teor. Appl..

[32] Liedl A (2019). A 3D imaging textural characterization of pyroclastic products from the 1538 AD Monte Nuovo eruption (Campi Flegrei, Italy). Lithos.

[33] Schepp LL (2020). Digital rock physics and laboratory considerations on a high-porosity volcanic rock. Sci. Rep..

[34] Leu L, Berg S, Enzmann F, Armstrong R, Kersten M (2014). Fast X-ray micro-tomography of multiphase flow in Berea sandstone: A sensitivity study on image processing. Transp. Porous Media.

[35] Balcewicz M (2021). Digital rock physics: A geological driven workflow for the segmentation of anisotropic Ruhr sandstone. Front. Earth Sci..

[36] Lavallée Y, Kendrick JE, Papale P (2021). A review of the physical and mechanical properties of volcanic rocks and magmas in the brittle and ductile regimes. Forecasting and Planning for Volcanic Hazards, Risks, and Disasters.

[37] Chiodini G, Pappalardo L, Aiuppa A, Caliro S (2015). The geological CO_2_ degassing history of a long-lived caldera. Geology.

[38] Piochi M, Cantucci B, Montegrossi G, Currenti G (2021). Hydrothermal alteration at the San Vito area of the Campi Flegrei geothermal system in Italy: Mineral review and geochemical modeling. Minerals.

[39] Gebauer S, Schmitt A, Pappalardo L, Stockli D, Lovera O (2014). Crystallization and eruption ages of Breccia Museo (Campi Flegrei caldera, Italy) plutonic clasts and their relation to the Campanian ignimbrite. Contrib. Mineral. Petrol..

[40] Houghton BF, Wilson CJN (1989). A vesicularity index for pyroclastic deposits. Bull. Volcanol..

[41] Goodfellow I, Bengio Y, Courville A (2016). Deep Learning.

[42] Ronneberger O, Fischer P, Brox T, Navab N, Hornegger J, Wells W, Frangi A (2015). U-Net: Convolutional networks for biomedical image segmentation. Medical Image Computing and Computer-Assisted Intervention—MICCAI 2015.

[43] Ledig, C. *et al*. *Photo-Realistic Single Image Super-Resolution Using a Generative Adversarial Network*. 10.48550/arXiv.1609.04802 (2017).

[44] He, K., Zhang, X., Ren, S. & Sun, J. *Deep Residual Learning for Image Recognition*. 10.48550/arXiv.1512.03385 (2015).

[45] Lim, B., Son, S., Kim, H., Nah, S. & Lee, K. M. *Enhanced Deep Residual Networks for Single Image Super-Resolution*. 10.48550/arXiv.1707.02921 (2017).

[46] Yu, J. *et al*. *Wide Activation for Efficient and Accurate Image Super-Resolution*. 10.48550/arXiv.1808.08718 (2018).

[47] Wang YD, Armstrong RT, Mostaghimi P (2019). Enhancing resolution of digital rock images with super resolution convolutional neural networks. J. Pet. Sci. Eng..

[48] Goodfellow, I. J. *et al*. *Generative Adversarial Networks*. 10.48550/arXiv.1406.2661 (2014).

[49] Mirza, M. & Osindero, S. *Conditional Generative Adversarial Nets*. 10.48550/arXiv.1411.1784 (2014).

[50] Isola, P., Zhu, J.-Y., Zhou, T. & Efros, A. A. *Image-to-Image Translation with Conditional Adversarial Networks*. 10.48550/arXiv.1611.07004 (2016).

[51] Anderson TI, Vega B, Kovscek AR (2020). Multimodal imaging and machine learning to enhance microscope images of shale. Comput. Geosci..

[52] Anderson TI, Vega B, McKinzie J, Aryana SA, Kovscek AR (2021). 2D-to-3D image translation of complex nanoporous volumes using generative networks. Sci. Rep..

[53] Zhu, J.-Y., Park, T., Isola, P. & Efros, A. A. *Unpaired Image-to-Image Translation Using Cycle-Consistent Adversarial Networks*. 10.48550/arXiv.1703.10593 (2017).

[54] Studholme C, Hill DLG, Hawkes DJ (1999). An overlap invariant entropy measure of 3D medical image alignment. Pattern Recognit..

[55] Wang Z, Bovik AC, Sheikh HR, Simoncelli EP (2004). Image quality assessment: From error visibility to structural similarity. IEEE Trans. Image Process..

[56] Otsu N (1979). A threshold selection method from gray-level histograms. IEEE Trans. Syst. Man Cybern..

[57] Giberti G, Yven B, Zamora M, Vanorio T, Zollo A, Capuano P, Corciulo M (2006). Database on laboratory measured data on physical properties of rocks of Campi Flegrei volcanic area (Italy). Geophysical Exploration of the Campi Flegrei (Southern Italy) Caldera’ Interiors: Data, Methods and Results.

[58] Heap MJ, Baud P, Meredith PG, Vinciguerra S, Reuschlé T (2014). The permeability and elastic moduli of tuff from Campi Flegrei, Italy: Implications for ground deformation modelling. Solid Earth.

[59] Montanaro C (2016). Experimental investigations on the explosivity of steamdriven eruptions: A case study of Solfatara volcano (Campi Flegrei). J. Geophys. Res. Solid Earth.

[60] Vanorio T, Prasad M, Nur A, Patella D (2002). Ultrasonic velocity measurements in volcanic rocks: Correlation with microtexture. Geophys. J. Int..

